# Influence of individual biological traits on GPS fix-loss errors in wild bird tracking

**DOI:** 10.1038/s41598-020-76455-x

**Published:** 2020-11-12

**Authors:** Ruth García-Jiménez, Antoni Margalida, Juan M. Pérez-García

**Affiliations:** 1 Department of Animal Science, Faculty of Life Sciences and Engineering, University of Lleida, 25198 Lleida, Spain; 2 Institute for Game and Wildlife Research, IREC (CSIC-UCLM-JCCM), Ronda de Toledo s/n, 13071 Ciudad Real, Spain; 3 Ecology Area, Department of Applied Biology, University Miguel Hernández, 03202 Elche, Spain

**Keywords:** Behavioural ecology, Behavioural methods

## Abstract

In recent decades, global positioning system (GPS) location data and satellite telemetry systems for data transmission have become fundamental in the study of basic ecological traits in wildlife biology. Evaluating GPS location errors is essential in assessing detailed information about the behaviour of an animal species such as migration, habitat selection, species distribution or foraging strategy. While many studies of the influence of environmental and technical factors on the fix errors of solar-powered GPS transmitters have been published, few studies have focussed on the performance of GPS systems in relation to a species’ biological traits. Here, we evaluate the possible effects of the biological traits of a large raptor on the frequency of lost fixes—the fix-loss rate (FLR). We analysed 95,686 records obtained from 20 Bearded Vultures *Gypaetus barbatus* tracked with 17 solar-powered satellite transmitters in the Pyrenees (Spain, France and Andorra), between 2006 and 2019 to evaluate the influence of biological, technical, and environmental factors on the fix-loss rate of transmitters. We show that combined effects of technical factors and the biological traits of birds explained 23% of the deviance observed. As expected, the transmitter usage time significantly increased errors in the fix-loss rate, although the flight activity of birds revealed an unexpected trade-off: the greater the proportion of fixes recorded from perched birds, the lower the FLR. This finding seems related with the fact that territorial and breeding birds spend significantly more time flying than non-territorial individuals. The fix success rate is apparently due to the interactions between a complex of factors. Non-territorial adults and subadults, males, and breeding individuals showed a significantly lower FLR than juveniles-immatures females, territorial birds or non-breeding individuals. Animal telemetry tracking studies should include error analyses before reaching any ecological conclusions or hypotheses about spatial distribution.

## Introduction

Obtaining a global positioning system (GPS) fix and the reliability of location data are primarily subject to satellite acquisition, a process mainly shaped by technical, environmental, and behavioural factors^1^. External factors such as GPS satellite geometry (satellite constellation), topography and land surface roughness, vegetation, fix interval (time lapse between successive fixes), or even GPS-tag position and orientation, all limit a transmitters’ ability to make contact with at least three satellites during a period of GPS activation^2–4^, causing GPS misconnections. Some authors have even observed: (1) an association between resource use, habitat selection, and fix-loss rate; and (2) interactions between animal behaviour and local habitat conditions which have to be considered particularly when assessing a species’ habitat use^4–8^. However, one of the biggest gaps in our understanding of GPS performance is related to species-specific behavioural effects. For example, the position of an individual animal changes the orientation of a receiver, and its performance. Some studies of large mammals have demonstrated that inactive animals have higher fix-loss rates and lower fix accuracy than active ones^1,4,9^. But very little is known about how, or to what extent, individual biological traits such as sex, age, size, territorial or breeding status, and their associated behaviour and ecology may affect satellite connection, fix-loss and location accuracy^10–13^. This kind of information is essential to properly interpret geolocation data and to draw useful conclusions regarding animal movement patterns or species behaviour.

During the last 40 years, Argos Platforms Transmitter Terminals (PTTs) have provided the world’s most commonly used tracking coverage technology for the remote study of free-ranging animal movements, mainly because of their integration of GPS fixes (i.e. satellite locations) with data transmission technologies (i.e. the Argos data transfer system), particularly from the mid-1990s when GPS receivers became able to record high-spatial-resolution tracking data^14–17^. However, the raw data registered through GPS-Argos telemetry still suffer from errors and biases (e.g. fix rate bias, fix-loss errors and spatial location errors) that must be considered to avoid drawing incorrect conclusions and making the wrong management recommendations^13,15,18^. These tracking problems are especially relevant for threatened species where reliable information is particularly important for reintroduction projects and conservation plans.

The endangered Bearded Vulture *Gypaetus barbatus* represents a good case study for assessing GPS fix-loss errors—measured in this study though the monthly fix-loss rate, FLR (for more details see “Methods” section). In the first instance, this species inhabits rugged mountain landscapes (in the Pyrenees, average home range kernel 90% varying between 63 km^2^ for territorial individuals to 11,600 km^2^ for non-territorial ones^19^) that allows the evaluation of the influence of abrupt topography on GPS fix-loss. Second, the long daylight hours and sunny climatic conditions favour at the same time flying behaviour and the charging of transmitter solar batteries. Third, the territorial behaviour of breeding individuals is very different to the behavioural pattern of non-territorial individuals, which fly over greater distances due to the lack of a nest site acting as a central foraging point^19,20^. Four, the changing seasonal and weather conditions in the Pyrenees allow the comparison of transmitter performance during different solar radiation conditions. Finally, Bearded Vultures are an endangered species (more specifically, classed by the BirdLife International 2017^21^ as vulnerable in Europe, and globally near threatened) for which accurate GPS data is important to improve management and conservation actions. The species is being reintroduced in several European countries, and GPS transmitter monitoring is one of the main tools used by managers and conservationists to assess its habitat use and reintroduction success^22^.

**Figure 1:**
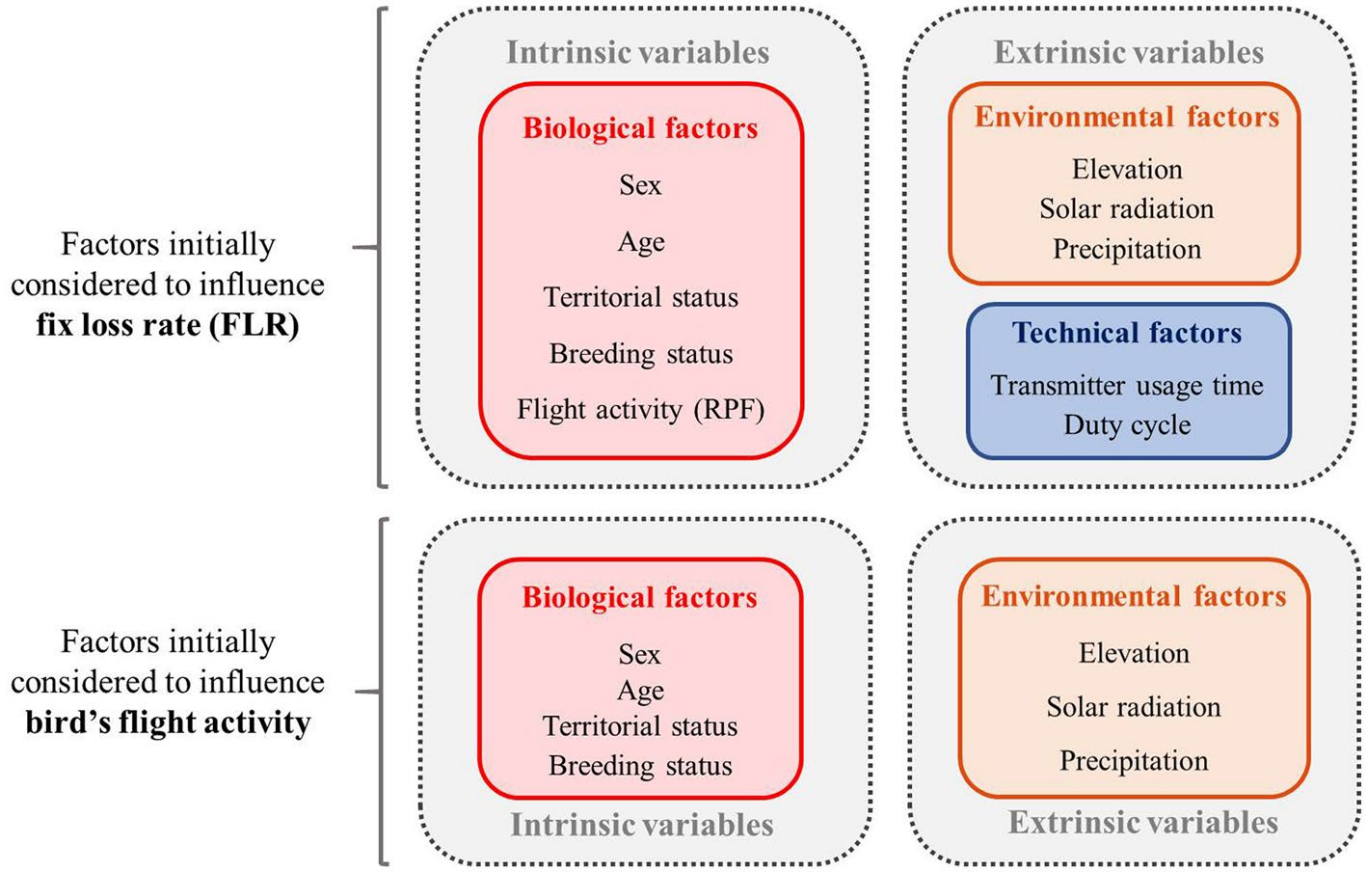
Factors considered to influence Fix Loss Rate (FLR) and bird’s flight activity (composed considering both monthly numbers of fix perched and fix in flight).

Technological improvements enabling the use of Argos GPS-lightweight PTTs (< 80 g) in marine mammals, birds, or even small animals up to 300 g^23,24^, have prompted new research into sources of GPS errors associated to wildlife telemetric tracking, especially when fix-loss rate is related to animal behaviour or habitat use. This study focuses on the biological, environmental, and technical factors affecting the fix-loss rate—either caused by GPS misconnections or battery undercharging—in Argos GPS PTTs. We considered specific biological traits of Bearded Vultures including sex, age, territorial and breeding status, and flight activity (derived from the monthly rates of fixes of perched and flying birds) as biological factors. Concurrently, we considered environmental variables including topographic altitude, surface solar radiation, and total precipitation, as well as technical factors considering the transmitter usage time and the duty cycle (i.e. fix recording scheduled regimes), as extrinsic factors. Afterwards, given the flying nature of our case study species and the effect that this kind of movement behaviour has showed over some technological characteristics of the GPS transmitters in previous studies^25^, we explore the influence of these biological and environmental variables over the flight activity trying to better understand this behavioural parameter and consequently its effect on the FLR (see Fig. 1). Based on previous solar-powered GPS tracking studies^13,25–27^, our hypothesis was that both FLR and flight activity of birds are strongly influenced either by specific biological traits and/or extrinsic factors, especially those related to technical factors. We hypothesized that individuals with greater flight activity (higher rates of fix in flight, RFF—reasonably assumed to be non-territorial individuals, who usually travel larger distances^19,20^), would be more exposed to direct solar radiation, thus present increased battery charging, and a lower FLR compared to territorial individuals. Considering extrinsic factors, weather conditions will affect fix reception success because periods with more daylight hours (i.e. summer, presenting the highest surface solar radiation and lowest total precipitation) also favour thermal conditions for flight, in contrast to winter, promoting thus birds’ flight activity. Topographic altitude will also probably affect FLR due to the challenging GPS connection in steep terrains. At the same time, it is expected that the transmitter usage time will negatively affect transmitter performance as a consequence of the decreasing battery and electronic system performance of the device.

## Results

A total of 95,686 location results from 20 Bearded Vultures tracked with 17 transmitters in the Pyrenees were recorded from 2006 until January 2019. Of these records: 32.6% were from females and 67.4% from males; 83.8% were from adults and 11.0% from subadults; 4.6% were from immatures and 0.6% from juveniles; 35.6% were from territorial birds and 64.4% from non-territorial ones.

### Fix-loss rate (FLR)

We found a substantial FLR variability showing significant differences between individual birds (Kruskal–Wallis, χ^2^ = 278.13, df = 18, p < 0.001) and also between individual PTTs (χ^2^ = 251.39, df = 15, p < 0.001). Five PTTs showed an FLR less than a 30%, seven showed FLRs of between 30 and 40%, one had an FLR of 48.2%, and the remaining five registered an FLR equal to or higher than 50% (Table 1). The FLR was highly variable at the individual level: seven birds had an FLR less than 30%; another seven showed FLRs between 30 and 40%; two ranged from 40 to 50%; and four showed an FLR higher than 50% (Table 1). However, FLR barely fluctuated between months, showing no significant differences over the year (Kruskal–Wallis, χ^2^ = 10.92, df = 11, p = 0.45), ranging between mean values of 0.31 ± 0.24 in May to 0.37 ± 0.23 in October.

**Table 1:** Basic biological traits and individual measures of fix loss rate (FLR) during a set period of time; rate of fix in flight (RFF) and rate of perched fixes (RPF) (mean ± SD for the monthly FLR, RPF and RFF individual values) for 20 birds tagged with 1770 g solar-powered Argos’ satellite transmitters (PTT/GPS Microwave Telemetry, Inc. Columbia, MD, USA) all bought in 2005–2008.

Individuals	PTT	Sex	Age (years)	Territorial status	FLR	Time period	Usage time (years)	RPF	RFF
Adrian	PTT1	M	4	T (2012–2016)	0.30 ± 0.15	05/2009–01/2019	9.8	0.72 ± 0.26	0.28 ± 0.26
Andreia	**PTT2**	H	≥ 7	T (2009)	0.29 ± 0.15	03/2009–09/2009	0.6	0.47 ± 0.32	0.52 ± 0.32
Pocholo	**PTT2**	M	≥ 7	NT	0.16 ± 0.14	07/2011–01/2019	7.6	0.75 ± 0.23	0.25 ± 0.23
Batín	PTT3	M	≥ 7	T (2008)	0.29 ± 0.15	05/2008–04/2015	7.0	0.50 ± 0.33	0.50 ± 0.33
Cabó	**PTT4**	H	≥ 7	T (2007)	0.47 ± 0.15	11/2007–08/2008	0.7	0.53 ± 0.37	0.47 ± 0.37
Sofia	**PTT4**	H	≥ 7	NT	0.34 ± 0.20	11/2008–05/2012	3.6	0.71 ± 0.29	0.29 ± 0.29
Dulantz	PTT5	M	6	NT	0.22 ± 0.19	04/2013–10/2014	1.5	0.64 ± 0.30	0.37 ± 0.30
Elisabeth	PTT6	H	18	NT	0.30 ± 0.21	03/2015–01/2018	2.9	0.77 ± 0.23	0.23 ± 0.23
Garrotxa	PTT7	H	5	T (2012)	0.40 ± 0.29	05/2008–06/2013	5.2	0.61 ± 0.31	0.38 ± 0.31
Gervàs	**PTT8**	H	≥ 7	T (2007)	0.28 ± 0.19	05/2007–04/2009	1.9	0.69 ± 0.28	0.31 ± 0.28
Min	**PTT8**	M	5	NT	0.56 ± 0.30*	05/2009–08/2017	8.4	0.73 ± 0.27	0.27 ± 0.28
Isaac	PTT9	M	5	NT	0.19 ± 0.16	11/2010–01/2014	3.2	0.70 ± 0.26	0.29 ± 0.26
Jairo	PTT10	H	4	T (2014)	0.32 ± 0.17	11/2010–06/2016	5.6	0.76 ± 0.27	0.24 ± 0.27
Morreres	PTT11	M	1	NT	0.28 ± 0.15	11/2007–09/2012	4.9	0.62 ± 0.31	0.37 ± 0.31
Nicky	PTT12	M	5	T (2011)	0.64 ± 0.30*	06/2009–05/2017	8.0	0.53 ± 0.34	0.47 ± 0.34
Noah	PTT13	H	≥ 7	NT	0.48 ± 0.13	04/2008–09/2008	0.5	0.84 ± 0.23	0.16 ± 0.23
Revilla	PTT14	H	5	NT	0.33 ± 0.17	04/2013–11/2013	0.6	0.87 ± 0.17	0.13 ± 0.17
Sasi	PTT15	M	1	NT	0.61 ± 0.24*	08/2007–06/2008	0.9	0.78 ± 0.24	0.22 ± 0.24
Subfli	PTT16	H	4	T (2012)	0.33 ± 0.17	05/2008–04/2012	4.0	0.72 ± 0.28	0.28 ± 0.28
Tossal	PTT17	H	≥ 7	T (2006)	0.74 ± 0.27*	11/2006–12/2006	0.1	0.82 ± 0.22	0.18 ± 0.22

In bold indicate transmitters (platform transmitter terminal, PTT) that were used on two different birds and * indicates when FLRs were equal or higher than 50%. For the territorial status (*T* territorial, *NT* non-territorial) the years of the beginning and ending (if any before 2019) are shown. The PTT FLRs were the same as the individual values of FLRs showed in this table, excepting for the case of the three PTTs that were reused: PTT2 presented a mean monthly FLR = 0.17 ± 0.14; PTT4 presented a mean monthly FLR = 0.36 ± 0.21 and PTT8 presented a mean monthly FLR = 0.50 ± 0.30*.

**Figure 2:**
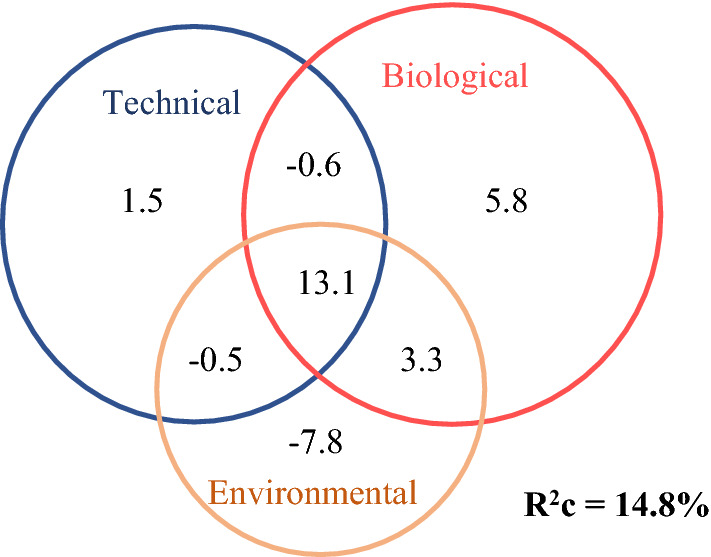
Conditional R^2^ partitions resulting from a partial regression analysis of 17 Microwave PTTs monthly fix-loss rates. Percentages of conditional R^2^ (deviance explained by the entire model, including both fixed and random effects) explained by each group of variables: *Technical* (PTT’s lifetime and duty cycle), *Biological* (rate of perched fixes, sex, age, territorial and breeding status), and *Environmental* (surface solar radiation, total precipitation, and topographic altitude) and by their interactions. The total conditional R^2^ of the model is also shown.

The total conditional R^2^ obtained from the GLMM built to evaluate the joint contribution of the biological, technical and environmental factors to the FLR was 0.148 (0.093 of the marginal R^2^ corresponding to the fixed effects + 0.055 of deviance corresponding to the random effects). The highest was provided by the sum of both technical factors (single effect of 1.5%) and biological traits (with the highest single retained effect of 5.8%) and its interactions with the other groups (− 0.6% shared between both groups, − 0.5% shared between technical factors and environmental factors, 3.3% shared between biological traits and environmental factors, and 13.1% resulting from the interaction of the three groups). Environmental factors retained a single effect of − 7.8% (Fig. 2).

Regarding the GLMMs results (Table 2), we found two models from the total selection that met the delta AIC < 2 criterion. The parameters influencing the efficient performance of the transmitters included all of the biological and technical variables tested. Non-territorial birds, males, and breeding individuals showed a significantly lower FLR than females, territorial birds or non-breeding vultures. The rate of perched fixes (RPF) showed a negative relationship with the FLR, while the PTT usage time exerted the opposite effect, so that the higher the RPF and—in parallel—the smaller the PTT usage time, the lower the FLR becomes. Indeed, longer duty cycles provoked also lower FLR, although this variable was only selected for one of the two final models selected. Regarding age, adults and subadults showed significantly lower FLRs. None of the environmental variables were included in the significant GLMMs eventually built. The partial effects of all the explanatory variables included at least in one of the two final models selected are shown in Supplementary Figure S1.

**Table 2:** Competing GLMMs to evaluate the influence of different biological traits and extrinsic factors (comprising both technical and environmental variables) on the fix loss rate (FLR).

Model	Factors	K	AIC	∆AIC	W
M1	T_PTT − RPF + Age + Sex + Territ + Br_S + (1|Indiv)	10	6929.6	0	0.725
M2	T_PTT – Dcycle − RPF + Age + Sex + Territ + Br_S + (1|Indiv)	11	6931.6	1.94	0.275

The individual (Indiv) was included as a random factor. We present the most parsimonious selected model with ΔAIC < 2. *K* total number of parameters (explanatory terms + random term + residual deviance), *AIC* corrected Akaike information criterion, *ΔAIC* difference between the AIC value for that model and the best model, *W* Akaike weight. Biological traits included: flight activity measured through the rate of fix perched (RPF), age (Age), territorial status (Territ), breeding season (Br_S) and sex (Sex). Technical variables were transmitter usage time (T_PTT) and duty cycle (Dcycle), and environmental variables were topographic altitude, surface solar radiation, and total precipitation.

### Flight activity

Considering all the data, we observed an average of 64.3 ± 20.0% rate of perched fixes (RPF) and 31.8 ± 16.5% rate of fixes in flight (RFF). Of the 20 Bearded Vultures tagged, 65.0% (n=13) showed a quite homogeneous flight activity pattern, their mean RPF ranging between 87.4 and 68.8%. Three individuals showed rates of 61.3–63.5% and the other four showed perched fix rates lower than 52.8%. Even so, three of the birds exhibited a higher monthly RFF than RPF (Table 1).

The RPFs ranged significantly between 70.4% in summer and 66.4% in winter (Kruskal–Wallis, χ^2^ = 21.12, df = 3, p < 0.001). The variables selected for the competing GLMMs influencing flight activity (Table 3) were territoriality, breeding status, age, and sex (although, the last two were not always included in the final models). Territorial and breeding individuals showed significantly lower RPFs than non-breeding and non-territorial ones. The mean RPF and RFF were 50.2 ± 25% and 39.3 ± 22.6% for territorial individuals and 72 ± 10.5% and 27.6 ± 10% for non-territorial birds, respectively. The environmental variables were not included in the final models. The partial effects of all the explanatory variables included at least in one of the four final models selected are shown in Supplementary Figure S2.

**Table 3:** Competing GLMMs for evaluating the influence of different biological traits and environmental variables on birds’ flight activity (computed as a weighted rate of perched fix).

Model	Factors	K	AIC	∆AIC	W
M1	Territ + Br_S + (1|Indiv)	4	7154.2	0.00	0.3
M2	Sex + Territ + Br_S + (1|Indiv)	5	7154.4	0.19	0.3
M3	Age + Sex + Territ + Br_S + (1|Indiv)	8	7155.1	0.90	0.2
M4	Age + Territ + Br_S + (1|Indiv)	7	7155.4	1.17	0.2

The individual (Indiv) was included as a random factor. We present the most parsimonious selected models with ΔAIC < 2. *K* total number of parameters (explanatory terms + random term + residual deviance), *AIC* corrected Akaike Information Criterion, *ΔAIC* difference between the AIC value for that model and the best model, *W* Akaike weights. Biological traits included: age (Age), territorial status (Territ), breeding season (Br_S), and sex (Sex). Environmental variables were topographic altitude, surface solar radiation, and total precipitation, but none was selected for the final models.

## Discussion

Studies of movement ecology often suffer from lost geolocation information due to: (1) technical glitches such as insufficient battery power; (2) environmental factors such as the roughness of the terrain (i.e. the conjunction of vegetation and topography^28^) or changing climatological parameters; and (3) intrinsic factors (i.e. biological traits) such as the behaviour of individuals^25,29^. Our findings point out that a combination of technical variables and biological traits gave the best explanation of maximum deviance (22.6%), suggesting that these two groups of factors have a much greater influence on the monthly FLR than environmental factors. This was also one of the main conclusions achieved by Hofman et al.^27^, in a study where they gathered information of 167 projects deployed on 62 species in 142 study areas worldwide through some questionnaires with the aim of assessing the performance of satellite telemetry units (predominantly collars) tracking terrestrial wildlife. Concretely, they found out that the transmitter performance was strongly influenced by unit and species characteristics while environmental conditions increased the variability, influencing the transmitters’ technique effectiveness. Concurrently, we propose that it could be that technical and biological variables already gather part of the deviance explained by the environmental factors. Such is the case for the breeding status, a parameter directly related to time of year, seasonality, solar radiation, and daylight duration in addition to its biological significance for the species. Another technical variable that affects the FLR, the transmitter battery level, is also related to solar energy availability, and hence to the time of year (through the seasonal variations in solar irradiance received by the device)^25,26^. Battery power limits the time for the transmitter to search satellites to obtain a location and so influence in the number of satellites acquired for the process^1^, however it is a parameter only available in the newer GPS models. In this respect, the logger generation, transmitters’ manufacturers, and data receiving system—all of the three uniform parameters for our study case given that all the 17 devices were Microwave solar-powered Argos-GPS bought between 2005 and 2008—are also important technical variables that need to be considered when studying GPS accuracy and location errors^25,29,30^. On the other hand, our results show that individual flight activity could be one of the most influential factors determining the fix performance of a device. Contrary to previous studies^19,36^ and our initial hypothesis, the greater the proportion of perched fixes, the lower is the resulting FLR. One possible explanation for this observation could be related to the difficulty of satellite acquisition while a bird is flying, as has been noted for moving animals in various mammal studies^29,31,32^, perhaps because of changes in the position and orientation of the GPS transmitter. Our findings also confirmed that longer duty cycles (of 2 h compared with those of 1 h) produced lower FLR, probably associated with the fact that more intense duty cycles increase the transmitter energy consumption and consequently reduce the device usage time^33^. In fact, Silva et al.^25^ suggested that FLR due to poor GDOP (when Geometric Dilution of Precision limits the transmitter to contact with enough satellites to produce a fix) increased when the birds moved. Nevertheless—considering that the time to obtain a fix increase in dynamic versus static conditions—longer duty cycles (of 2 h compared to 30 s and 15 min interval times) would produce higher fix loss rates while flying, but the opposite situation could happen while the birds are perched, when the length of the fix interval is not so relevant. In addition, as it was predicted, the FLR increases with transmitter usage (as happened in^27,34^), a relevant information considering that the mean usage time for our PTTs was 5.34 ± 3.03 years (n = 14).

Given the number of studies which point to landscape structure as an important driver of the FLR^2,35^, we expected the topographic altitude as a variable influencing FLR. However, our monthly-scale analysis could have diluted the effect of this environmental variable and a complementary shorter time-scale FLR study (e.g. daily or hourly) may show a higher influence of this specific variable on the fix loss errors. Notwithstanding these uncertainties, our analyses of the possible effects of Bearded Vulture biological traits on the FLR constitutes a novel approach to the better understanding of the treatment of PTT locations. All the biological variables tested in this study influenced the RPF and also significantly affected the fix reception success. Interestingly, non-territorial Bearded Vultures travel further and later in the daylight than territorial birds^20^, but exhibit significantly higher RPFs (72 ± 10.5% for non-territorial individuals versus 50.2 ± 25% for territorial birds). At the same time, breeding and territorial adults showed lower FLRs even if they spent less time perched than non-breeding, non-territorial and younger individuals. Probably their daily activity related to parental duties (nest-building, territorial defence, and foraging) results in increased flight activity and a higher proportion of their time spent flying, even if the distances covered are shorter than those of non-territorial birds^19,20,36^.

Our results showed an overall monthly mean FLR of 34.5 ± 24.72% ranging between a minimum of 3% and maximum of 100% (n = 17). This is lower than the values found for analogous transmitters by Silva et al.^25^, used on the same Pyrenean and Cantabrian population of Bearded Vultures (FLR = 0.40 ± 0.12), and those recorded by Soutullo et al.^24^, for lightweight Argos GPS transmitters used on Golden Eagles *Aquila chrysaetos* in a rocky cliff area in Eastern Spain (FLR = 0.45). In this latter study, breeding season also influenced the FLR (probably through seasonal effects), as was the case in our study. Nevertheless, our findings show the importance of understanding that significant variations in FLR may be due either to variations in individual bird behaviour or to variations in technical glitches affecting each PTT performance. Therefore, it should be expected that both biological and technical factors play a fundamental role in the correct performance of the GPS fix programming.

The significant differences in FLR between male and female birds are not easily explained from a behavioural and ecological perspective (mean values of 0.32 for males cf. 0.39 for females), even if non-territorial males do indeed exploit larger areas and fly over longer distances, as it is the case of territorial females^19,20^. The specific relationships between the biological traits of this species and RPF or RFF are clear, but even if their influence on the FLR is also obvious, it is more difficult to explain the effect of certain biological variables such as territoriality or sex on FLR. The fix success rate results are most likely due to a synergy between complex interactions; for instance, between flight height and terrain roughness, or between the availability of environmentally optimal flight conditions (which are also favorable for solar battery charging) linked to the likelihood of flight activity and the resulting associated increase in transmitter movement. In any case, it is clear from this study that biological factors such as sex, age, breeding and territorial status have particular effects on FLR and must be considered when studying fix error rates in other flying species (e.g. bird and bat species). Even considering the apparent limitations of working with a single species in a GPS fix loss error study, as it has been shown, our findings can be extrapolated to different medium and large-size animal populations and species. Moreover, technological improvements of materials and both hardware and software enhancements are leading to increasingly better transmitters’ performance with improved location accuracy and reduced FLRs. However, there are still many transmitters in use (in addition to the quite a few already developed) that present scheduled location duty cycles, data receiving Argos-GPS system and device manufacturers similar to the ones evaluated in this study, so these findings obtained remain relevant for long-term conservation studies.

Every animal telemetry tracking study should include an error analysis before reaching any ecological conclusions or hypotheses regarding spatial utilization, since the results can vary substantially depending on extrinsic factors such as GPS transmitter model, retrieval data system, PTT usage time, season, etc., or biological factors such as those analysed in this study. All of these changing elements can influence the data collected and lead to errors in interpreting patterns of movement. Fortunately, these kinds of tracking error, together with accuracy biases in the horizontal plane (x and y coordinates) are being addressed and overcome as transmitter technology improves, thus reducing the potential influence of tracking device shortcomings on the recording and interpretation of basic parameters regarding the spatial ecology of a species^26,29,30,37,38^.

## Methods

### Study area

We assessed the GPS fix loss errors resulting from Bearded Vultures studied in the Pyrenees, a steep mountainous region with maximum altitudes of 3400 m, located in the north of the Iberian Peninsula on the border between France and Spain. It includes three different bioclimatic areas (Montane, Sub-Alpine and Alpine) with average annual temperatures between 0 and 20 °C, and a four-season Mediterranean climate with seasonal weather conditions^39^.

### Study species

The Bearded Vulture is a territorial, cliff-nesting vulture specialized in feeding on the bones of medium size ungulates^40^. In common with other avian scavengers it exploits thermal and orographic updrafts to use the least energy as possible when foraging. It is an endangered species now only found in certain mountainous areas of Europe, Asia, and Africa^21,41^. In the Pyrenees, the spatial ecology of this species has been studied since the 1980s’, originally using conventional VHF radio tracking^42–44^ and more recently with the solar-powered Argos or GSM data recovery system with GPS-PTTs^19,20,45^.

### Tracking and data origin

Between 2006 and 2019, twenty Bearded Vultures were tagged with 17 different 70 g solar-powered Argos’ satellite transmitters (PTT/GPS Microwave Telemetry, Inc. Columbia, MD, USA, all of the 2005–2008 logger generation)—three of which were reused on new individuals—attached to the bird’s back with a breakaway thoracic junction stitched with cotton thread harness made of 0.64 cm Teflon ribbon (Bally Ribbon Mills, Bally, PA, USA) (for further details see^15^). The usage time of the transmitters was 5.34 ± 3.03 years (n = 14) on average. To compute this mean value for the three reused PTT, we summed the time usage of each peer of individuals using the same PTT, and for the rest of the PTTs, we excluded the records corresponding to the birds dead on the field (n = 3) since their transmitters could not be recovered and the reason for stopping fix recording was unlikely related with technical causes. We only considered the records of individuals whose PTTs stopped working properly, accounting times from the moment the PTTs were turned on until the moment we stopped receiving location data (see Supplementary Table S1). All of the transmitters were programmed to report hourly GPS fixes between 04:00 and 22:00 UTC hours each day (manufacturer estimated error ± 18 m), except for two individuals whose PTTs transmitted every 2 h. Regarding the biological factors: (1) age of individuals were assigned to four different age classes according to plumage characteristics: juvenile (1 year old); immature (2–3 years old); subadult (4–5 years old); and adult (> 6 years old) (for details see^19,20^); (2) sex was determined by molecular analysis of blood samples (PCR amplification of the CHD-W gene as described in^46^); (3) territoriality was described as territorial or non-territorial individuals, depending on their breeding behaviour^20^; (4) breeding season was defined either as *breeding period* (1st January to 31st July) or *non-breeding period* (1st August–31st December)^15^; and (5) flight activity was defined according to Silva et al.^25^ by the complementary rates of perched fixes (RPF, calculated from monthly fixes with speeds slower than 1.39 m/s) and fix in flight (RFF, calculated from monthly fixes with speeds equal or faster than 1.39 m/s) (Tables 1 and 2). Regarding the extrinsic factors: for technical variables, (1) we accounted for the device usage time and (2) duty cycle (as mentioned, of 1 or 2 h depending on the individual) and for environmental variables, (3) topographic altitudes were obtained using a Digital Elevation Model (ASTER Global DEM, 1 arc-second spatial resolution); and (4) surface solar radiation and (5) total precipitation were obtained from an interim full-daily at surface forecast (European Centre for Medium-Range Weather Forecasts, 0.75° each 3 h). Monthly means of all three parameters were calculated using the Movebank Env-DATA track data annotation service^47,48^ (Fig. 1).

### Data processing and statistical analysis

The fix-loss rate (FLR) used in this study was calculated as a monthly value for each individual consisting of the proportion of days per month on which no fixes were recorded. We evaluated the effects of both biological and extrinsic factors (including both technical and environmental variables) on the performance of the 17 transmitters represented by monthly FLRs computed as the number of days per month on which no data were collected, divided by the total number of days on which data were scheduled to be collected. We generated a data set of monthly observations (n = 889), each with its own FLR. Since we reused three of the 17 transmitters to track the movement pattern of 20 birds, we needed to distinguish between two different levels when computing mean FLRs: the PTT/transmitter level and the individual level. For instance, the PTT usage time depends directly on the transmitter but variables related with the biological traits depend uniquely on the individual.

At first, we examined the FLR with some non-parametric explorative analyses to evaluate possible differences among these two levels and to evaluate the influence of the month on the FLR yearly distribution. Secondly, we grouped all the predictor variables: (1) age, sex, breeding and territorial status, and RPF (this latter describing flight activity) as biological factors; (2) PTT usage time per month and duty cycle as the technical factors; and (3) monthly means of topographic altitude, surface solar radiation, and total precipitation as environmental factors (see Fig. 1).

Thirdly, we performed a deviance partitioning analysis^49^ to evaluate the effect on FLR of the single and joint contributions of each of the three groups of variables comparing by basic algebra the percentage of the explained conditional R^2^ of each of the best generalized linear mixed models (GLMMs)^50^ built including the aforementioned biological, technical and environmental factors as fixed factors (where applicable) and the individual as a random factor. Thus, we built seven separate GLMMs to evaluate: (1) the single contribution of the biological factors, (2) the single contribution of the technical factor; (3) the single contribution of the environmental factor; (4) the joint contribution of the biological and technical factors; (5) the joint contribution of the biological and environmental factors; (6) the joint contribution of the technical and environmental factors; and (7) the joint contribution of the biological, technical and environmental factors (see more details about how to perform a deviance partitioning analysis in^51^). These analyses were computed using R statistical software^52^ version 3.6.2. For the GLMMs, we applied the “glmer” function of the “lme4” R package^53^ with a binomial error distribution and logit-link function. All the deviance explained by the different groups of variables was expressed in percentages when we referred to the deviance partitioning results.

Fourthly, to determinate the significant variables influencing the FLR, we constructed the full model with all of the biological, technical and environmental variables as fixed factors and the individual as a random factor considering again a binomial error distribution and logit-link function, made a model selection using Akaike’s Information Criterion (AIC^54^), and chose the best models with a delta AIC < 2 (Fig. 1).

And fifthly, to better understand the individual flying behaviour and how it could affect FLR, we analysed the influence of all of the same biological and environmental factors on the flight activity of the birds. For this analysis, a weighted RPF (wRFP) was created combining the monthly number of perched fix and monthly number of fix in flight (see Zuur et al.^55^ for applying binomial generalized models for proportions). Thus, we modelled wRPF using a GLMM (binomial error distribution and logit-link function) with all the biological and environmental variables as fixed factors and the individual as a random factor, and then selected models giving delta AIC < 2 (Fig. 1). Technical factors were not included as predictors in this model because of their obvious absence of influence over the flight activity of the birds.

For all the mixed models built in this study, the relative contributions of the fixed and random factors to R^2^ were estimated with the “r.squaredGLMM” function from the package “MuMIn”^56^. We also reviewed for the variance inflation factors (VIF) for all the predictor variables at the first stages of the GLMMs building using the “car” package^57^ to assess collinearity (accepted VIF values < 3). In fact, we firstly considered season (defined as yearly quarterly periods i.e.: winter, from January to March; spring, from April to June; summer, from July to September; and fall, from October to December) and month for all the GLMMs’ analyses, but they were finally excluded because of their high correlation with breeding season and surface solar radiation. All continuous variables were standardized and centred before modelling using the “scale” R function and all of the non-parametric analyses were performed after checking for the absence of a normal distribution.

Tracking data are inherently auto-correlated, although if fixes are taken infrequently enough so as to be longer than the autocorrelation timescale of the data, data can be considered independent, especially for animals that move long distances in short periods of time^5,58^. This is the case for our study species in this study, which present minimum duty cycles of 1 h (see also^19^).

### Ethics statement

All the work was conducted in accordance with relevant national and international guidelines, and conforms to all legal requirements. Captures and blood sample collection were carried out in compliance with the Ethical Principles in Animal Research. Thus, protocols, amendments and other resources were conducted in accordance to the guidelines approved by the Catalan Autonomous Government (Generalitat de Catalunya) following the R.D.1201/2005 (10 October 2005, BOE 21 October 2005) of the Ministry of Presidency of Spain. All experimental protocols were approved by the Catalan Autonomous Government and MAGRAMA (References 15.546 and 25.306).

## Supplementary information

Supplementary Information.

## Data Availability

The datasets used and analyzed during the current study are available from AM on reasonable request.
