# Supplementary material for: Influence of individual biological traits on GPS fix-loss errors in wild bird tracking
Source: Sci Rep. 2020 Nov 12;10:19621. doi: 10.1038/s41598-020-76455-x (PMC7665197; doi:10.1038/s41598-020-76455-x)

**Supplementary Material**

**Influence of individual biological traits on GPS fix-loss errors in wild bird tracking**

**Ruth García-Jiménez^1*^, Antoni Margalida^2*^ and Juan M. Pérez-García^3*^**

^1^ Department of Animal Science, Faculty of Life Sciences and Engineering, University of Lleida, 25198 Lleida, Spain.

^2^ Institute for Game and Wildlife Research, IREC (CSIC-UCLM-JCCM), Ronda de Toledo s/n, 13071 Ciudad Real, Spain.

^3^ Ecology Area, Department of Applied Biology, University Miguel Hernández, 03202 Elche, Spain.

***Corresponding authors:**

**Ruth García-Jiménez**

E-mail: ruth.garciajimenez@gmail.com

**Antoni Margalida**

E-mail: a.margalida@csic.es

**Juan Manuel Pérez-García**

E-mail: juanmapg@gmail.com

**Table S1.** Year of capture and PTT activity status of 20 Pyrenean Bearded Vultures tagged by 70 g solar-powered Argos’ satellite transmitters (PTT/GPS Microwave Telemetry, Inc. Columbia, MD, USA) between 2006 and 2019. In bold type the three transmitters (platform transmitter terminal, PTT) used for two different birds and * for the PTTs that couldn’t be used to compute the mean usage time value.

| Individuals | PTT | Year captured | PTT activity until 2019 (ending year) |
| --- | --- | --- | --- |
| Adrian | PTT1 | 2009 | working |
| Andreia | **PTT2** | 2009 | dead (2009) |
| Pocholo | **PTT2** | 2011 | working |
| Batín | PTT3 | 2008 | stopped transmitting (2015) |
| Cabó | **PTT4** | 2007 | dead (2008) |
| Sofia | **PTT4** | 2008 | stopped transmitting (2012) |
| Dulantz | PTT5* | 2013 | dead (2014) |
| Elisabeth | PTT6 | 2014 | stopped transmitting (2018) |
| Garrotxa | PTT7 | 2008 | stopped transmitting (2013) |
| Gervàs | **PTT8** | 2007 | dead (2009) |
| Min | **PTT8** | 2010 | stopped transmitting (2017) |
| Isaac | PTT9 | 2010 | stopped transmitting (2014) |
| Jairo | PTT10 | 2009 | stopped transmitting (2016) |
| Morreres | PTT11 | 2007 | stopped transmitting (2012) |
| Nicky | PTT12 | 2009 | stopped transmitting (2017) |
| Noah | PTT13 | 2008 | stopped transmitting (2008) |
| Revilla | PTT14* | 2013 | dead (2013) |
| Sasi | PTT15 | 2007 | stopped transmitting (2008) |
| Subfli | PTT16 | 2008 | stopped transmitting (2012) |
| Tossal | PTT17* | 2006 | unknown (2006) |

**Figure S1:** Partial effects of all the explanatory variables included at least in one of the competing GLMMs built to evaluate the influence of different biological traits and extrinsic factors (comprising both technical and environmental variables) on the fix loss rate (FLR). The most parsimonious models were selected using Akaike’s Information Criterion (AIC^34^) and met the delta AIC < 2 criterion. In these models, the biological, technical and environmental variables were included as fixed factors and the individual as a random factor. All continuous variables were centred and standardized before modelling. All the plots were obtained using R statistical software^51^ version 3.6.2.


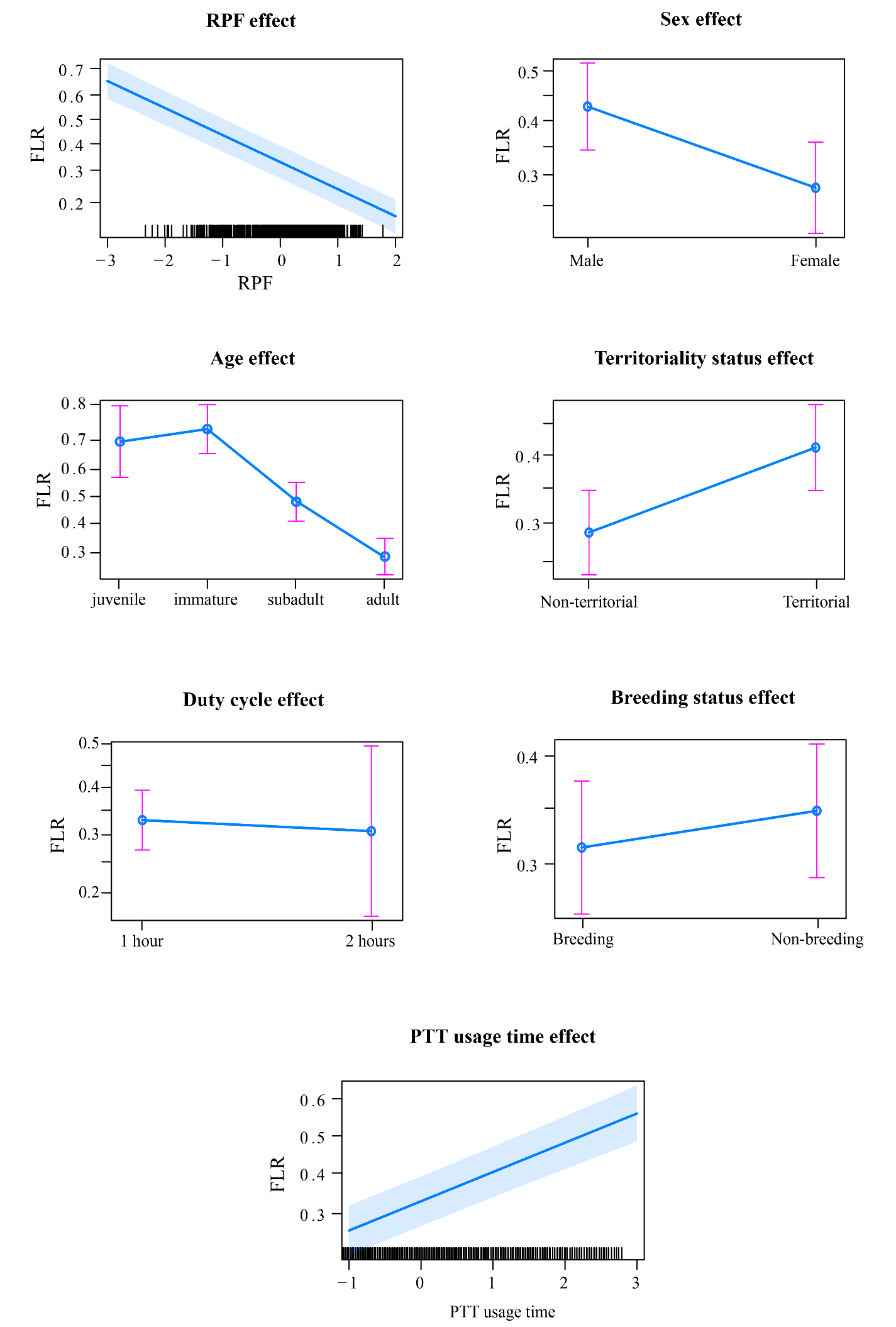


**Figure S2:** Partial effects of all the explanatory variables included at least in one of the competing GLMMs built to evaluate the influence of different biological traits and environmental variables on birds’ flight activity. For this analysis, the response variable was measured through a weighted rate of perched fix (wRFP) created by combining the monthly number of perched fix and monthly number of fix in flight. The most parsimonious models were selected using Akaike’s Information Criterion (AIC^34^) and met the delta AIC < 2 criterion. To build the models, the biological and environmental variables were included as fixed factors and the individual as a random factor. All continuous variables were centred and standardized before modelling. All the plots were obtained using R statistical software^51^ version 3.6.2.


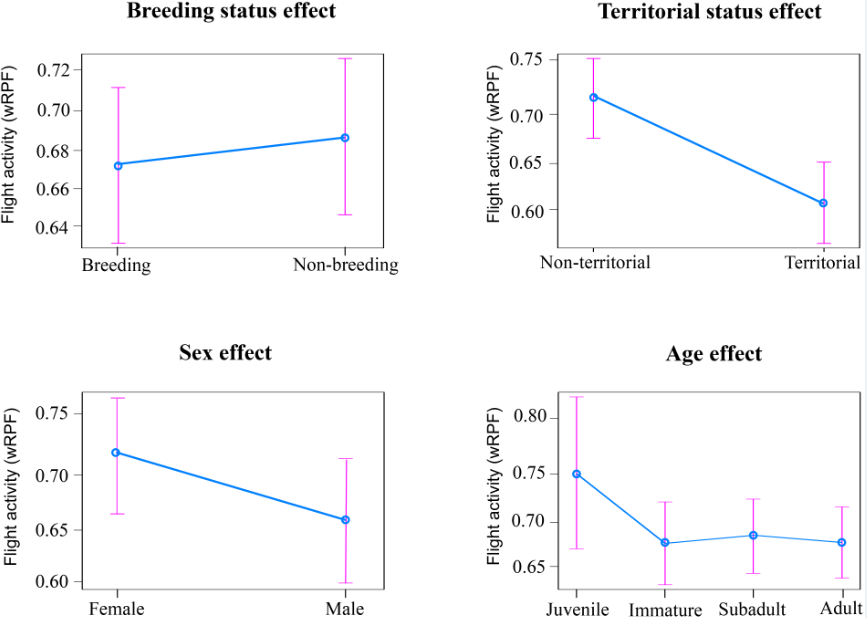

Supplement: Supplementary file 1 — Supplementary Information. [file 41598_2020_76455_MOESM1_ESM.docx]
